# New reconstruction of the *Wiwaxia* scleritome, with data from Chengjiang juveniles

**DOI:** 10.1038/srep14810

**Published:** 2015-10-07

**Authors:** Zhifei Zhang, Martin R. Smith, Degan Shu

**Affiliations:** 1 Early Life Institute, State Key Laboratory of Continental Dynamics, Northwest University, Xi’an, 710069, P. R. China; 2 Department of Earth Sciences, University of Cambridge, Downing Street, Cambridge, CB2 3EQ, UK

## Abstract

Wiwaxiids are a problematic group of scale-covered lophotrochozoans known from Cambrian Stages 3–5. Their imbricating dorsal scleritome of leaf-like scales has prompted comparison with various annelids and molluscs, and has been used as a template to reconstruct the articulation pattern of isolated Small Shelly Fossils. The first articulated specimens of *Wiwaxia* from the Cambrian Stage 3 Chengjiang *Konservat-Lagerstätte* show that the *Wiwaxia* scleritome comprised nine equivalent transverse rows associated with outgrowths of soft tissue, but did not possess a separate zone of anterior sclerites. This serial construction is fundamentally incompatible with the circumferential disposition of sclerites in early molluscs, but does closely resemble the armature of certain annelids. A deep homology with the annelid scleritome must be reconciled with *Wiwaxia*’s mollusc-like mouthparts and foot; together these point to a deep phylogenetic position, close to the common ancestor of annelids and molluscs.

The distinctive mid-Cambrian organism *Wiwaxia* is best known for its stalked carbonaceous sclerites, which together comprise an imbricated dorsal scleritome. Articulated scleritomes have previously been reported from five localities spanning 15 million years^1^,^2^,^3^,^4^,^5^,^6^. The constitution of the *Wiwaxia* scleritome is remarkably conserved, notwithstanding species-level variety in sclerite proportions and orientation. An anterior zone of sclerites is followed by eight transverse rows across the body, with morphologies varying consistently according to location: ventrolateral sclerites are sickle-shaped; lower-lateral sclerites are oval; upper-lateral sclerites are rounded and symmetrical, and dorsal sclerites are asymmetric. In addition, mature specimens – those longer than a centimetre – exhibit twin series of erratically arranged dorsal spines. The anterior body region seemingly corresponds to a distinct zone of rounded sclerites^4^, although the exact relationship of these anterior sclerites to the transverse rows is unclear.

The construction of the sclerites and scleritome represents important, if ambiguous, evidence with respect to the phylogenetic affiliation of *Wiwaxia*. The sclerites bear the distinctive signature of microvillar secretion – an internal microstructural fabric of long, narrow chambers – which assigns *Wiwaxia* to the lophotrochozoan total group^7^. (The persistent occurrence of these striations through the full length of intact sclerites distinguishes them from the parallel striations reported in certain ecdysozoan sclerites^8^,^9^). Beyond this, the interpretation of the scleritome is more ambiguous. Some authors^10^,^11^ have favoured an annelid analogue, but others^12^,^13^ have emphasized similarities with aculiferan molluscs – accommodating the conspicuously mollusc-like nature of the *Wiwaxia* foot (as observed in a small number of specimens) and feeding apparatus^12^,^14^.

Here we report a new species of *Wiwaxia* based on articulated specimens from the Cambrian Stage 3 Chengjiang *Lagerstätte*. Post-mortem enrolment and soft tissue preservation in these fossils, in combination with critical *W. corrugata* material from the Burgess Shale, allows a timely re-evaluation of the *Wiwaxia* scleritome, and expounds the scleritome’s implications for the affinity of this confounding taxon.

## Material and Methods

Five new *Wiwaxia* specimens, each comprising part and counterpart, have been collected from Chengjiang by the Early Life Institute working team, and deposited in the Early Life Institute and Department of Geology, Northwest University, Xi’an, China (Prefix: ELI). These complete, articulated specimens represent organisms in various states of enrolment, preserved at various orientations to the plane of splitting. Sclerites and mouthparts are represented by regions with a deep purple to black colouration, and in the best cases correspond to an intact layer of carbon. Regions corresponding to soft tissue are coated with rust-coloured framboids, apparently arising through the oxidation of pyrite.

Burgess Shale specimens of *W. corrugata* are deposited in the Smithsonian Institution National Museum of Natural History (NMNH), Washington DC, and the Royal Ontario Museum (ROM), Toronto, and represent unweathered carbonaceous compression fossils associated with diagenetic aluminosilicate films^15^.

### Systematic Palaeontology

This published work and the nomenclatural acts it contains have been registered in Zoobank: http://zoobank.org/Referencesurn:lsid:zoobank.org:pub:1B5E0AE5-2FB2-4EFF-B35B-8293D919DEE8

Family Wiwaxiidae Walcott, 1911 (nom. corr. Howell, 1962)

Genus *Wiwaxia* Walcott, 1911

#### Emended diagnosis

Ovoid body bearing nine transverse rows of ribbed carbonaceous sclerites, arranged in bundles and directed towards the posterior. Anteriormost sclerite row terminal. Sclerites comprising narrow root and wide blade, and incorporating narrow internal longitudinal chambers. Sclerite morphology varying consistently across each transverse row; medial sclerites rounded, ventro-lateral sclerites elongate and curved, usually with pointed tip. Dorsal surface of adults often with elongate spinose sclerites. Ventral surface comprising unarmoured ‘foot’. Toothed feeding apparatus comprising two to three rows of curved carbonaceous teeth arranged on tongue-like supporting apparatus.

*Wiwaxia papilio* sp. nov.

*LSID.* urn:lsid:zoobank.org:act:E5A1A18C-D087-4FA1-AC60-A0CE65603E9B

#### Derivation of name

Papilio (Latin), butterfly, reflecting the butterfly-like arrangement of the fans of sclerites.

#### Holotype

**Figure 1:**
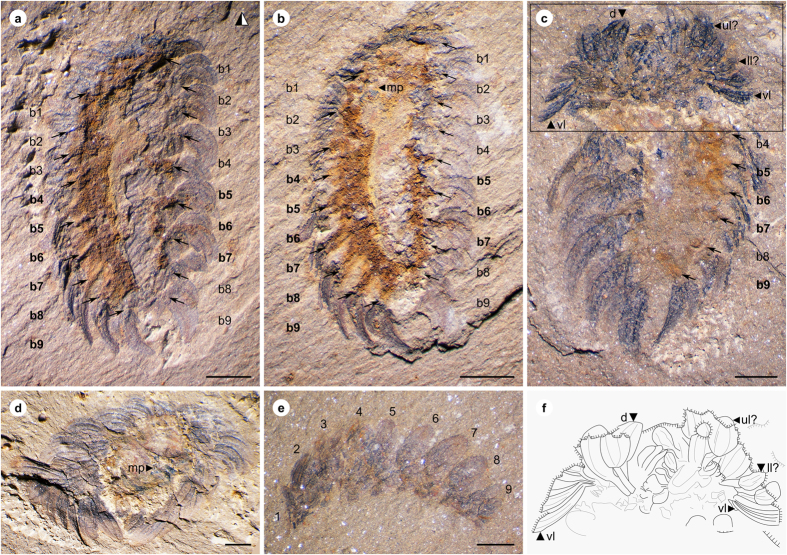
*Wiwaxia papilio* sp. nov. from Chengjiang. (**a,b**), ELI-W001, Holotype, part (horizontally mirrored) and counterpart, ventral view showing bundles of sclerites (b1–b9) associated with rust-coloured outgrowths of soft tissue (bold-face lettering), showing mouthparts (mp); (**c**), ELI-W004, ventral view, with anterior body curved up to display anterior sclerite zone; sclerites in anterior row express morphologies of ventrolateral (vl), lower lateral (ll), upper lateral (ul) and dorsal (**d**) sclerites; interpretative sketch in (**f**); (**d**), ELI-W003, anterior view showing mouthparts (mp); (**e**), ELI-W005, lateral view, illustrating anterior sclerite row and eight subsequent dorsal sclerites (1–9). Scale bars: 1 mm. Z. Zhang and M. Smith created the images.

ELI-W001 (Fig. 1a–b), an almost complete dorsoventral specimen preserving mouthparts and soft tissue.

#### Paratypes

**Figure 2:**
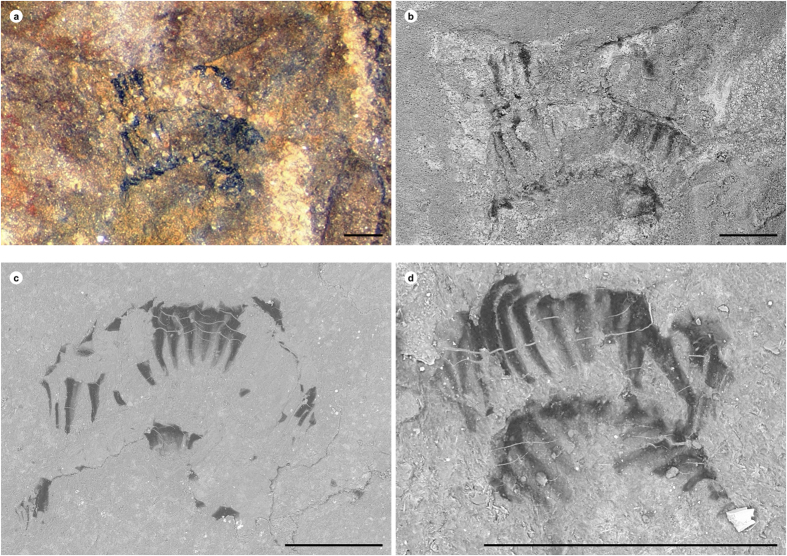
Mouthparts of *Wiwaxia*. (**a,b**), ELI-W003, *Wiwaxia papilio* sp. nov., optical (**a**) and backscatter electron (**b**) images; (**c,d**) *Wiwaxia corrugata* from the Burgess Shale; (**c**), NMNH 277890; (**d**), NMNH 271947. Scale bars = 250 μm. Z. Zhang and M. Smith created the images.

ELI-W002–ELI-W005 (Figs 1c–e, 2a,b).

#### Stratigraphic setting

Specimens were collected from the yellowish-green to greyish-green mudstones of the Chengjiang *Lagerstätte* at the Jianshan Section in Haikou, Kunming. Other taxa recovered from this site include the early agnathan *Haikouichthys*^16^ and the echinoderm-like vetulocystids^17^.

#### Diagnosis

**Figure 3:**
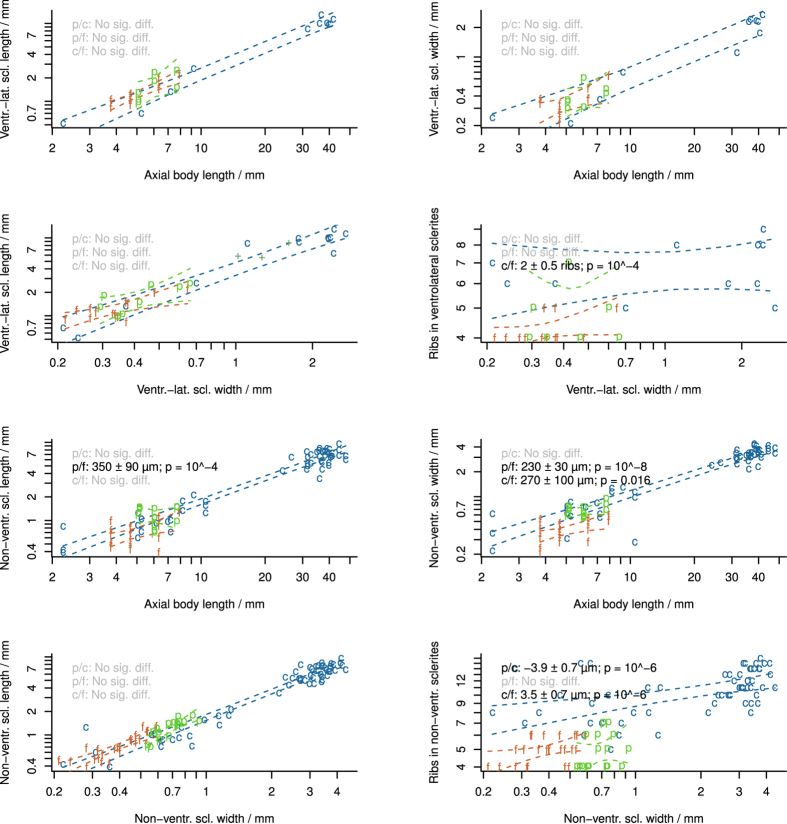
Summary of sclerite measurements in *Wiwaxia* species. c *W. corrugata*; f *W. foliosa*; p, *W. papilio* sp. nov.; + , isolated Chengjiang sclerites. Dashed lines denote 95% the confidence envelope of regression lines. Within each panel, regression line gradients are not significantly different. Panel legends report significant pairwise differences in the location of the y-intercept. M. Smith created the images. For source data and statistical code, see Supplementary Datasets.

Single order of widely spaced sclerite ribs (4–6 ribs on sclerites 500–1000 μm in length). Non-ventrolateral sclerites long and wide relative to body length (Fig. 3).

#### Remarks

The examined material resembles juvenile specimens of *Wiwaxia corrugata* in terms of its overall body size, the form of its mouthparts, the relatively large size of dorsal sclerites, the broad yet short ventrolateral sclerites, and the absence of dorsal spines. Adult specimens are conceivably represented by the larger isolated sclerites that have also been reported from Chengjiang^18^ (Fig. 3), though this material is difficult to exclude from other *Wiwaxia* species. *W. papilio* sp. nov. is distinguished from *W. corrugata* based on the low number ribs on its sclerites; detailed comparison with other species is hampered by the shortage of comparative material^1^,^5^.

#### Description

**Figure 4:**
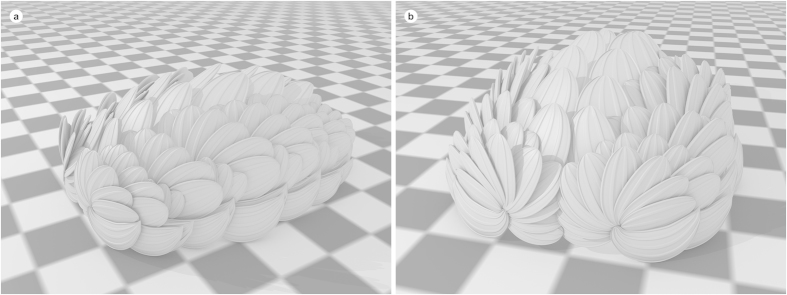
Reconstruction of *Wiwaxia papilio* sp. nov. (a) lateral view, showing arrangement of transverse rows; (b), frontal view, showing fan-like arrangement of anterior sclerites. Checkers = 1 mm. M. Smith and Z. Zhang created the images.

The articulated specimens of *W. papilio* sp. nov. (Figs 1 and 4) are 5–8 mm long, and exhibit the arrangement of sclerites typical of juvenile *Wiwaxia* specimens: an anterior region of sclerites followed by eight further transverse rows, with no dorsal spines. Ventrolateral sclerites are siculate, whereas other sclerites are rounded and about twice as long as broad. (The limited preservation of the dorsal surface precludes a detailed description of the dorsal most sclerites.) Each ventro-lateral sclerite fully overlaps its posterior neighbour (*per* ref. 2, but *contra* ref. 12). Two specimens preserve mouthparts with two to three rows of carbonaceous teeth (Fig. 2a,b). As the morphology of individual teeth is indistinct, it is not clear whether or not small lateral teeth are present; otherwise, no substantive differences from *W. corrugata* (Fig. 2c,d) are evident.

**Figure 5:**
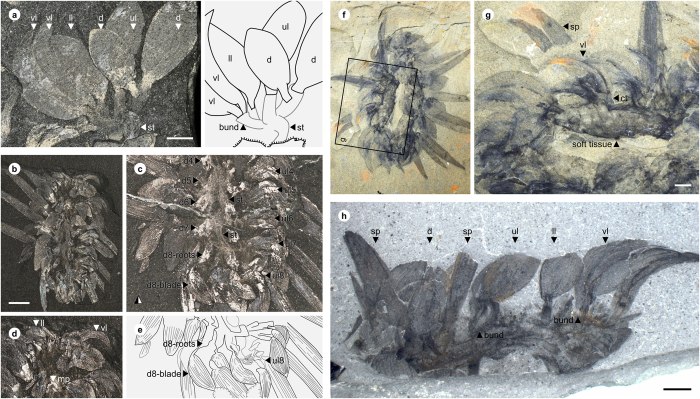
*Wiwaxia corrugata* from the Burgess Shale. (**a**) NMNH 199948, partial row of sclerites and associated connective tissue; (**b–e**), ROM 57707, connective tissue associated with bundles of dorsal and upper lateral sclerites; images courtesy Jean-Bernard Caron; c is horizontally mirrored counterpart of specimen in b (**f–g**), ROM 61511, outgrowth of connective tissue associated with bundle of ventrolateral sclerites; (**h**), NMNH 199953; transverse row of sclerites articulated by connective tissue. M. Smith created the images. *Abbreviations:* bund, bundling of sclerites; ct, connective tissue; d, dorsal sclerite; d4–8, bundles of dorsal sclerites; ll, lower lateral sclerite; mp, mouthpart; sp, spine; ul, upper lateral sclerite; ul4–8, bundles of upper lateral sclerites; vl, ventrolateral sclerite. Scale bars = 2 mm.

The anterior row of sclerites is made up of two bilaterally-paired rosettes, across which the morphology of sclerites varies in the same fashion as it does elsewhere on the body: the most ventral sclerites are siculate, whereas the more dorsal sclerites are rounded and occasionally asymmetric (Figs 1c and 4b). Siculate sclerites also form part of the anterior row of Burgess Shale specimens of *Wiwaxia corrugata* (e.g. Fig. 5b,d), occurring at the lateral edges of the scleritome but not skirting the front of the animal (*contra* ref. 4). As such, the anterior sclerites do not form a distinct zone of the scleritome, but represent a (ninth) transverse row of sclerites.

The ventral surface of the fossils is represented by an iron-rich region that we interpret as soft tissue in a position dorsal to the foot. Bundles of sclerites insert into lateral projections of this iron-rich region (Fig. 1a–c). Sclerite bundles are anchored in equivalent projections in *W. corrugata* (Fig. 5a–g; see ref. 12), where equivalent projections are connected by transverse bands of connective tissue (Fig. 5h); these themselves are embedded in the soft tissue of the organism (Fig. 5h).

## Discussion

### Distribution of *Wiwaxia*

This report represents the first occurrence of articulated *Wiwaxia* in the shallow water communities represented by the Chengjiang fauna. The five new specimens are all less than a centimetre long and lack spines; by analogy with *W. corrugata*, they represent juveniles. The only evidence of adult *Wiwaxia* individuals at Chengjiang comes from a single assemblage of ventro-lateral sclerites, corresponding in size to those of adult *W. corrugata*^18^; in contrast, there are a hundred adult *Wiwaxia* in the Burgess Shale for every five juveniles^12^. This reflects a more general scarcity of adult *Wiwaxia* specimens in shallow-water settings. Bedding-surface fossils from the Buchava, Hongjingshao and Kaili formations exclusively correspond to juvenile size ranges and morphologies^2^,^3^,^19^. (These localities, like Chengjiang, preserve shallow-water communities; in the case of Kaili, shallow-water taxa were washed into deeper waters before burial^19^,^20^,^21^,^22^,^23^).

In contrast, deep-water settings are replete with adult *Wiwaxia*. The deep water Tsinghsutung (=Qingxudong) Formation contains disarticulated sclerites that correspond to the size range of sclerites in adult *Wiwaxia corrugata*, and includes elongate sclerites that conceivably represent spines^3^,^24^. The Spence Shale and Sinsk Biota, which were deposited below storm wave base^25^,^26^, contains articulated and disarticulated sclerites belonging to *Wiwaxia* adults^1^,^6^,^27^. And in the Burgess Shale, adult *Wiwaxia* are present in great abundance at the deeper water localities on Fossil Ridge and Mount Stephen^4^,^12^,^28^,^29^ but have not yet been found in the shallow-water Marble Canyon locality^30^.

*Wiwaxia* juveniles occur in almost all geographic and ecological settings^31^,^32^, perhaps reflecting planktonic larval dispersal^33^. The rarity of adult specimens in shallow waters may therefore represent failure to reach maturity in these environments – whether through active migration to deeper water, or through accentuated predation pressure on adult organisms.

### Phylogenetic implications of scleritome constitution

Since the discovery of the first articulated specimens^10^, sclerite disposition has played a central role in determining *Wiwaxia*’s biological affinity.

One obvious analogue to *Wiwaxia* sclerites are the conspicuous dorsal scales (elytra) of aphroditid and polynoid annelids^10^,^34^ – but these fleshy outgrowths are not secreted by microvilli, so cannot be equivalent to *Wiwaxia* sclerites^4^,^11^. The modified paleal chaetae of chrysopetalid annelids represent a more promising analogue^11^; as with *Wiwaxia* sclerites, chrysopetalid paleae occur in a series of bundles or fans across a transverse rows^35^,^36^, and indeed sclerite morphology even varies from siculate lateral sclerites to more symmetrical dorsal sclerites^37^. This correspondence also rings true on the level of sclerite construction: chrysopetalid paleae, like *Wiwaxia* sclerites, comprise a proximal root and a broad distal blade, and on a more superficial level may exhibit ribs, a granular ornament, and a distal prong (cf. ref 32).

Despite this compelling similarity, there is a fundamental objection to a chrysopetalid affinity: chrysopetalids are fundamentally derived crown-group annelids^38^,^39^,^40^,^41^,^42^, whereas *Wiwaxia* lacks key synapomorphies such as biramous parapodia, palps and aciculae and thus belongs outside the annelid crown group^7^,^42^,^43^,^44^. Equally problematic is the location of the ventral mouthparts in *Wiwaxia* beneath the second or third sclerite row: this is difficult to reconcile with the anterior position of the annelid prostomium. As such, the detailed similarity between *Wiwaxia* sclerites and those of chrysopetalids must be attributed to convergent evolution.

Could the molluscs provide a more reasonable analogue for the *Wiwaxia* scleritome? Of the extant molluscs, only aculiferans (=Polyplacophora + Aplacophora) bear comparable sclerites. Polyplacophoran sclerites exhibit crystalline cores that are surrounded by a thin cuticular layer with a microvillar texture^45^,^46^, which is conceivably homologous (at a deep level) to the sclerites of *Wiwaxia*^12^ – but polyplacophoran sclerites are arranged in concentric zones rather than transverse rows, and exhibit a broadly quincuncial disposition^47^ rather than occurring in bundles. Aplacophoran molluscs do exhibit transverse rows of dorsal sclerites at early developmental stages^13^,^48^ – but stem-group aplacophorans resemble polyplacophorans^49^,^50^, meaning that *Wiwaxia* would have to represent a surprisingly early and extremely derived aplacophoran that retained larval features to adulthood and developed a precise sclerite organization unseen in modern representatives^12^. As such, no living mollusc provides a convincing analogue for *Wiwaxia*’s scleritome.

Although the detailed construction of the *Wiwaxia* scleritome has no precise equivalent in modern or fossil groups, this is not to say that it does not share homologies at a deeper level. Carbonaceous sclerites are secreted by microvilli in bryozoans, brachiopods, molluscs and annelids^46^,^51^,^52^, and are likely homologous across Lophotrochozoa^53^. The iterated arrangement of *Wiwaxia* sclerites is paralleled by basal annelids, and possibly brachiopods^54^,^55^,^56^. Finally, the bundling of sclerites in *Wiwaxia* could conceivably foreshadow the neuropodial and notopodial bundles observed in crown-group annelids. Whilst it is conceivable that the iterated nature of the *Wiwaxia* scleritome arose convergently, we prefer to assume homology in the absence of clear evidence to the contrary.

The broadly annelid-like serial construction of the *Wiwaxia* scleritome must be balanced against the conspicuously mollusc-like nature of its mouthparts and foot^12^,^14^,^57^,^58^. An equivalent paradox is represented in *Hallucigenia*, which bears onychophoran-like claws alongside cycloneuralian-like mouthparts; this is resolved if cycloneuralian-like mouthparts occurred in the common ancestor of onychophorans and cycloneuralians^59^. As the affinity of *Wiwaxia* is less well established, there is more than one way to reconcile its molluscan and annelidan features. Either annelid-like sclerite rows or molluscan mouthparts may have been present in the common ancestor of annelids and molluscs and been retained for some time in the stem lineages of each phylum.

**Figure 6:**
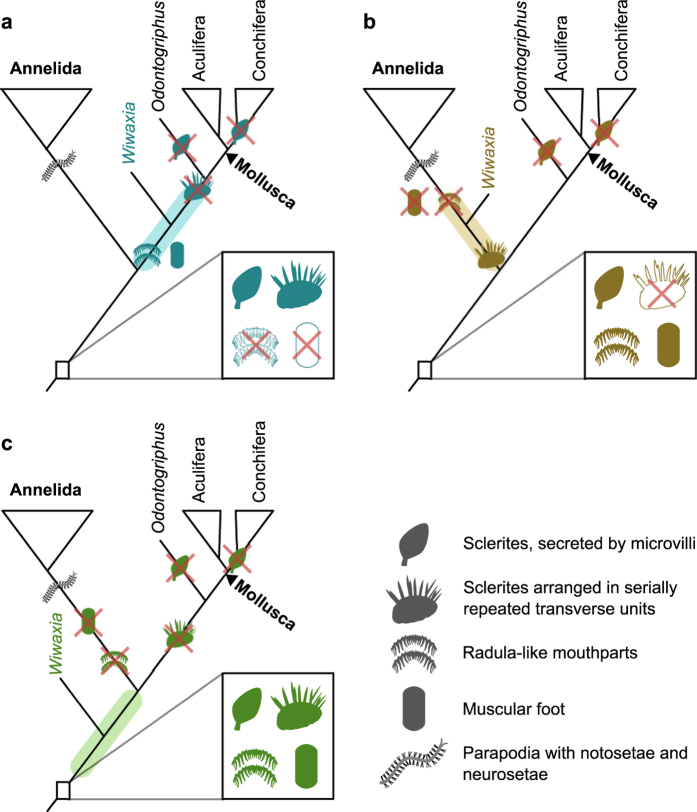
Possible affinities of *Wiwaxia*. (**a**), in molluscan stem group; (**b**), in annelid stem group; (**c**), in stem group to Mollusca + Annelida. Boxes denote implied morphology of deep ancestors of Annelida + Mollusca; branches are illustrated with implied gains or losses of characters. M. Smith and Z. Zhang created the images.

If *Wiwaxia* is a stem-group mollusc (Fig. 6a), a scleritome of iterated rows was conceivably ancestral to annelids and molluscs, and was later rearranged into the circumferential format of aculiferans. If *Wiwaxia* is a stem-annelid (Fig. 6b), a muscular foot and radula-like mouthparts are ancestral to molluscs and annelids, with a serially arranged scleritome unique to the annelid stem and ultimately leading to metamerism and full segmentation in the crown group. Under this arrangement, annelids replaced their ancestrally mollusc-like mouthparts with an independently-derived and non-homologous^14^ jaw, just as onychophorans replaced their cycloneuralian-like mouthparts with independently-derived jaws^59^. The third possibility is that *Wiwaxia* falls in the stem lineage of Mollusca + Annelida. Under this scenario, both phyla exhibit a loss or overprinting of primitive features: the foot and mouthparts in annelids, the transverse sclerite arrangement in molluscs.

Ultimately, a more complete record of early Lophotrochozoan evolution is needed before the polarity of these distinctive characters can be resolved. But whatever the exact phylogenetic position of *Wiwaxia*, it clearly diverged before the modern phyla had attained their distinctive body plans, and thus represents a valuable proxy for the common ancestor of molluscs and annelids.

## Additional Information

**How to cite this article**: Zhang, Z. *et al.* New reconstruction of the *Wiwaxia* scleritome, with data from Chengjiang juveniles. *Sci. Rep.* **5**, 14810; doi: 10.1038/srep14810 (2015).

## Supplementary Material

Supplementary Dataset 1

Supplementary Dataset 2

Supplementary Dataset 3

Supplementary Dataset 4
