# Publisher Correction: New reconstruction of the *Wiwaxia* scleritome, with data from Chengjiang juveniles

**DOI:** 10.1038/s41598-023-48303-1

**Published:** 2023-12-04

**Authors:** Zhifei Zhang, Martin R. Smith, Degan Shu

**Affiliations:** 1 State Key Laboratory of Continental Dynamics, Early Life Institute, Northwest University, Xi’an, 710069 P. R. China; 2 Department of Earth Sciences, University of Cambridge, Downing Street, Cambridge, CB2 3EQ UK

Correction to: *Scientific Reports* 10.1038/srep14810, published online 07 October 2015

This Article contains errors. A Supplementary Dataset file, which was included with the initial submission, was omitted from the original version of this Article. The omitted Supplementary Information file is linked to this correction notice.

### Supplementary Information

Supplementary Information.

