# Supplementary figures and images for: Publisher Correction: New reconstruction of the Wiwaxia scleritome, with data from Chengjiang juveniles
Source: Sci Rep. 2023 Dec 4;13:21346. doi: 10.1038/s41598-023-48303-1 (PMC10696083; doi:10.1038/s41598-023-48303-1)

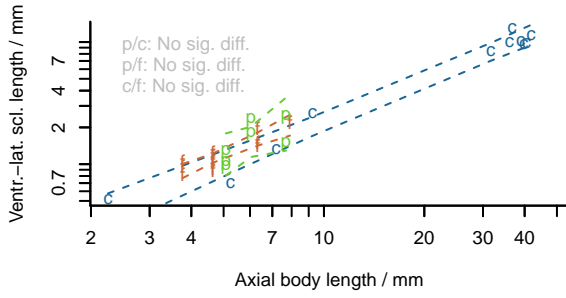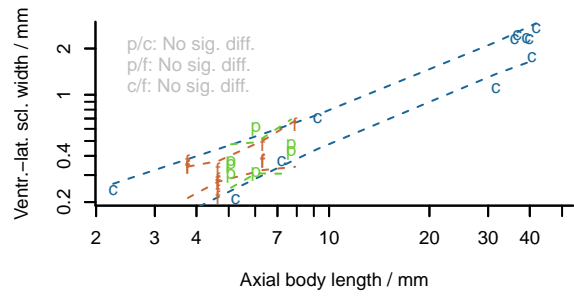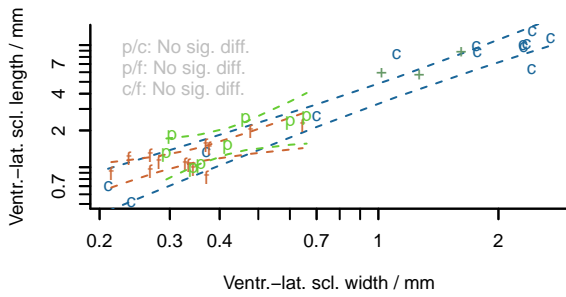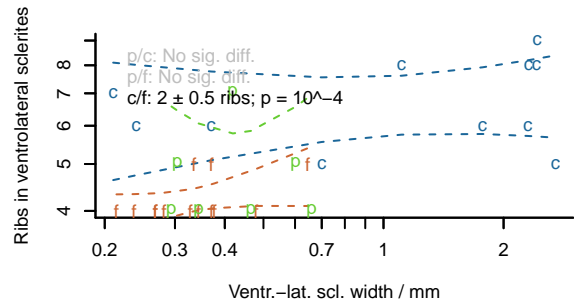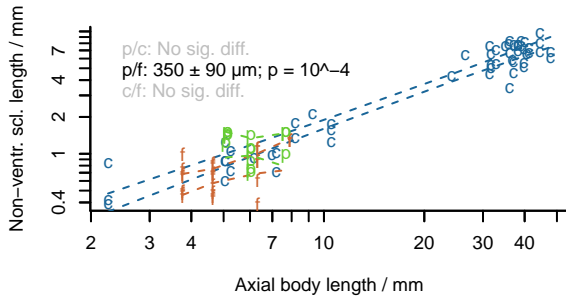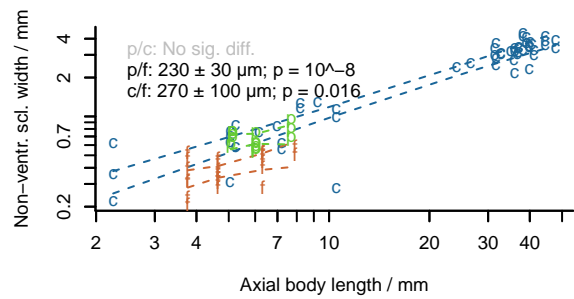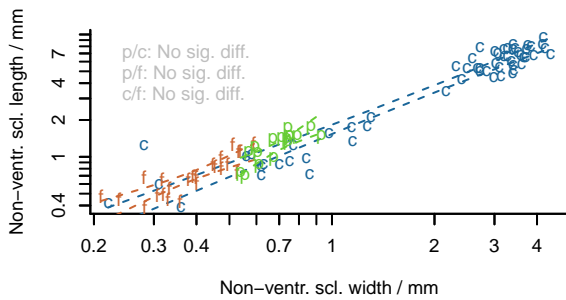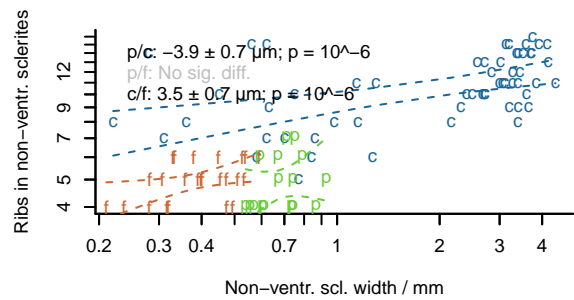

Supplement: Supplementary file 1 — Supplementary Information. [file 41598_2023_48303_MOESM1_ESM.zip › Wiwaxia_regress.pdf]
